# Performance of Graphene/Polydimethylsiloxane Surfaces against *S. aureus* and *P. aeruginosa* Single- and Dual-Species Biofilms

**DOI:** 10.3390/nano12030355

**Published:** 2022-01-22

**Authors:** Isabel M. Oliveira, Marisa Gomes, Luciana C. Gomes, Manuel F. R. Pereira, Olívia S. G. P. Soares, Filipe J. Mergulhão

**Affiliations:** 1 LEPABE—Laboratory for Process Engineering, Environment, Biotechnology and Energy, Faculty of Engineering, University of Porto, Rua Dr. Roberto Frias, 4200-465 Porto, Portugal; imoliveira@fe.up.pt (I.M.O.); marisagomes@fe.up.pt (M.G.); luciana.gomes@fe.up.pt (L.C.G.); 2 LSRE–LCM—Laboratory of Separation and Reaction Engineering–Laboratory of Catalysis and Materials, Faculty of Engineering, University of Porto, Rua Dr. Roberto Frias, 4200-465 Porto, Portugal; fpereira@fe.up.pt

**Keywords:** graphene, polydimethylsiloxane, *Staphylococcus aureus*, *Pseudomonas aeruginosa*, antibiofilm activity, implantable medical devices

## Abstract

The increasing incidence of implant-associated infections has prompted the development of effective strategies to prevent biofilm formation on these devices. In this work, pristine graphene nanoplatelet/polydimethylsiloxane (GNP/PDMS) surfaces containing different GNP loadings (1, 2, 3, 4, and 5 wt%) were produced and evaluated on their ability to mitigate biofilm development. After GNP loading optimization, the most promising surface was tested against single- and dual-species biofilms of *Staphylococcus aureus* and *Pseudomonas aeruginosa*. The antibiofilm activity of GNP/PDMS surfaces was determined by the quantification of total, viable, culturable, and viable but nonculturable (VBNC) cells, as well as by confocal laser scanning microscopy (CLSM). Results showed that 5 wt% GNP loading reduced the number of total (57%), viable (69%), culturable (55%), and VBNC cells (85%) of *S. aureus* biofilms compared to PDMS. A decrease of 25% in total cells and about 52% in viable, culturable, and VBNC cells was observed for *P. aeruginosa* biofilms. Dual-species biofilms demonstrated higher resistance to the antimicrobial activity of GNP surfaces, with lower biofilm cell reductions (of up to 29% when compared to single-species biofilms). Still, the effectiveness of these surfaces in suppressing single- and dual-species biofilm formation was confirmed by CLSM analysis, where a decrease in biofilm biovolume (83% for *S. aureus* biofilms and 42% for *P. aeruginosa* and dual-species biofilms) and thickness (on average 72%) was obtained. Overall, these results showed that pristine GNPs dispersed into the PDMS matrix were able to inhibit biofilm growth, being a starting point for the fabrication of novel surface coatings based on functionalized GNP/PDMS composites.

## 1. Introduction

Implantable medical devices (IMDs) play an active role in the therapy of different medical conditions, enhancing the quality of life [1,2]. Although they are extremely successful in supporting or even replacing damaged body organs, IMDs (e.g., cardiac implantable devices, hemodialyzers, urinary or central venous catheters, contact lenses, artificial breasts, and orthodontal and orthopedic prosthetics) carry the risk of inducing future infections, seriously affecting the patients’ health and even endangering their lives [3,4,5]. Implant-associated infections (IAIs) present a high incidence, corresponding to 60–70% of the nosocomial infections reported each year in the United States [6], and are responsible for a severe burden on healthcare systems and high economic costs [7,8].

IMDs are prone to bacterial adhesion and, consequently, biofilm formation, contributing to the persistence and spread of infection [9]. The implants act as foreign materials in the human body, enabling the colonization of several microbial species that are not eliminated by the host’s innate immune system. The predominant microorganisms identified as colonizers of IMDs include *Staphylococcus epidermidis* and *Staphylococcus aureus*, which account for 50–60% of IAIs [10]. However, depending on the type and location of the implant, other species can be found, namely *Escherichia coli*, *Pseudomonas aeruginosa*, *Enterococcus* spp., *Candida* spp., and *Klebsiella pneumoniae* [11,12]. Once adhered to the implant surface, the microorganisms form either single- or multi-species biofilms, which are typically less susceptible to antimicrobial treatment [13,14].

To overcome the limitations noted above, it is crucial to develop new strategies that specifically reduce the adhesion and growth of microorganisms [15]. Currently, one of the most promising antimicrobial strategies is the development of antifouling polymeric nanomaterials. These systems include the release of antimicrobial agents (e.g., metals and biocides), contact-killing materials (e.g., antimicrobial peptides), matrix disruptive agents (e.g., enzymes and cationic chelators), or a combination of these approaches [5,16,17]. Although there are promising strategies to mitigate IAIs, their application as a coating in IMDs is still limited by the low biocompatibility, the resistance phenomena, the toxicity that originates from the coatings, and the loss of antimicrobial properties over time [5,18].

Due to its remarkable chemical and physical properties, graphene has emerged as a novel material with relevant applications in the biomedical field [19]. This nanomaterial, in its pure form, is characterized by a single-layer sheet of sp^2^-hybridized carbon atoms with a honeycomb structure [20], and provides multiple advantages: it is easily renewable, easy to prepare and functionalize, and possesses a large surface area, high stability in the physiological environment, and unique mechanical strength [21]. Due to their outstanding antimicrobial activity, graphene-based materials (e.g., graphene nanoplatelets, graphene nanosheets, graphene oxide, and reduced graphene oxide) have been extensively studied for application as coatings/surfaces for biomedical devices [22,23]. However, it is worth noting the use of functionalized graphene-based materials for other applications, including biosensing and bioimaging, gene therapy, tissue engineering, and drug delivery [23,24]. For instance, the use of graphene-based composites as a support to release antimicrobial agents for wound dressing applications has been described [25,26]. The good biocompatibility of graphene, which has been improved through functionalization, clearly contributes to its high demand in the biomedical field [22,27].

The antibacterial activity of graphene-based materials involves physical and chemical mechanisms. The most usual antibacterial activity occurs upon direct contact to the bacterial membrane by the sharp edges of graphene sheets, or wrapping and trapping bacterial membranes by the nanosheets [28]. The chemical damage is associated with the oxidative stress originated by the production of molecules known as reactive oxygen species (ROS) [29].

The antimicrobial and anti-adhesive activity of graphene and its derivatives against implant-associated pathogens have been widely addressed, with significant inhibition of biofilm formation or even reduction of mature biofilms [23,30,31,32]. Despite the interesting results using graphene-based surfaces to prevent bacterial biofilm formation, the use of non-functionalized graphene as a coating for IMDs is still poorly explored and documented [22,23,29]. In addition, most of the studies reporting the use of pristine graphene tend to combine it with other materials with expected synergetic effects, such as silver nanoparticles [33,34], cadmium sulfide [35], titanium [36], magnetite [37], or chitosan [32], hindering the in-depth understanding of the effective antibiofilm performance of graphene alone. At the same time, there are a scarce number of studies reporting the employment of graphene to enhance the antibacterial properties of polydimethylsiloxane (PDMS) [38,39,40,41], a polymer belonging to the group of silicone elastomers with high applicability in IMDs [42,43].

Taking this evidence into account, the primary objective of this study was to optimize the graphene nanoplatelets (GNPs) loading of PDMS surfaces and evaluate the performance of these composites in the mitigation of single- and dual-species biofilms of *S. aureus* and *P. aeruginosa*—two common colonizers of IMDs. As far as we know, this is the first work reporting the antimicrobial activity of pristine GNPs against multi-species biofilms. Furthermore, there are no previous studies addressing the activity of pristine GNPs while incorporated into a polymeric surface against biofilms of the two specific bacterial species under study—*S. aureus* and *P. aeruginosa*.

## 2. Materials and Methods

**Figure 1:**
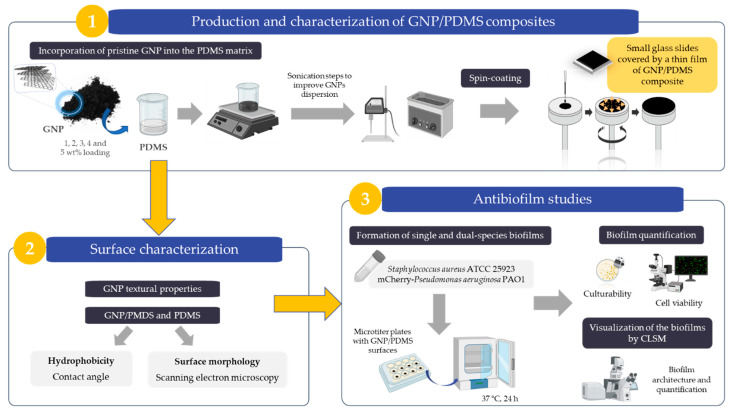
Representative scheme of the experimental tasks performed within the scope of this work.

Figure 1 presents the flowchart of the experimental work fully described in the upcoming sections.

### 2.1. Production of GNP/PDMS Composites

To prepare GNP/PDMS surfaces, commercially available GNPs aggregates (Alfa Aesar, Thermo Fisher Scientific, Erlenbachweg, Germany), PDMS elastomer (Sylgard 184 Part A, Dow Corning, Midland, MI, USA), and curing agent (Sylgard 184 Part B, Dow Corning) were used.

The GNP/PDMS composites were produced through a bulk mixing process by incorporation of GNPs into the PDMS base elastomer (Part A) as detailed by Vagos et al. [44]. The GNPs were first incorporated at different loadings (1, 2, 3, 4, and 5 wt%) to determine the most efficient GNP load to decrease bacterial biofilm formation. To improve the GNP dispersion, the mixture was stirred for 30 min at 500 rpm and then sonicated (Hielscher UP400S, at 200 W and 12 kHz) for 60 min. After that, the composites were kept in an ultrasound bath (Selecta Ultrasons, Lisbon, Tecnilab, Portugal) for 30 min to remove the remaining air bubbles. At that point, the curing agent (Part B) was added to the elastomer/GNP mixture (in an A:B ratio of 10:1) and gently shaken. The GNP composites were placed as a thin layer on top of glass slides (1 x 1 cm, Vidraria Lousada, Lda, Lousada, Portugal) through spin coating (Spin150 PolosTM, Caribbean, The Netherlands) for 1 min with a 500 rpm ramp to 6000 rpm. Likewise, PDMS surfaces were produced as control [45].

### 2.2. Surface Characterization

#### 2.2.1. GNP Textural Properties

Nitrogen adsorption-desorption isotherms were obtained using a Quantachrome NOVA 4200e multi-station equipment (Quantachrome Instruments, Boynton Beach, FL, USA) at −196 °C. These isotherms were used to assess the textural properties of the GNP sample after degasification at 120 °C for 3 h under vacuum. The surface area of the GNP sample was obtained according to the Brunauer–Emmett–Teller method (*S*_BET_), and the total pore volume (*V*_p_) was obtained from the amount of N_2_ adsorption at a relative pressure *p*/*p*_0_ of 0.99 [46]. The external surface area (S_meso_) and micropore volume (V_micro_) were also acquired applying the t-method.

#### 2.2.2. GNP/PMDS and PDMS Hydrophobicity

The contact angles of PDMS and GNP/PDMS surfaces at 1, 2, 3, 4, and 5 wt% GNP were obtained through the sessile drop method (OCA 15 Plus, Dataphysics, Germany). Contact angles with three pure liquids—water, formaldehyde, and α-bromonaphthalene—were estimated to determine the surface tension elements of the target surfaces. The surface tension components of the noted liquids (I) were collected from the literature [47]. Surface hydrophobicity was then assessed by the method of van Oss et al. [48,49,50]. In this method, the hydrophobicity level of a surface (*i*) is defined as the free energy of interaction between two elements of that surface immersed in water (*w*)-∆*G_iwi_*. If the interaction between the two elements is greater than the interaction of each element with water (∆*G_iwi_* < 0 mJ m^−2^), the surface is hydrophobic. Contrarily, if ∆*G_iwi_* > 0 mJ m^−2^, the material is considered hydrophilic. The value of ∆*G_iwi_* was obtained from the surface tension components of the interacting elements in conformity with Equation (1):

(1) $$\Delta G_{iwi}=-2{\left(\sqrt{\gamma_{i}^{LW}}-\sqrt{\gamma_{w}^{LW}}\right)}^{2}+4\left(\sqrt{\gamma_{i}^{+}\gamma_{w}^{-}}+\sqrt{\gamma_{i}^{-}\gamma_{w}^{+}}-\sqrt{\gamma_{i}^{+}\gamma_{i}^{-}}-\sqrt{\gamma_{w}^{+}\gamma_{w}^{-}}\right),$$

where *γ* ^*LW*^ corresponds to Lifshitz–van der Waals element of the surface free energy, and *γ* ^+^ and *γ* ^−^ are the electron acceptor and electron donor parameters, respectively, of the Lewis acid-base component (*γ ^AB^*), with γ *^AB^* =$\;2\sqrt{\;\gamma^{+}\;\gamma^{-}}$.

The surface tension components were determined by the simultaneous solving of three equations similar to Equation (2):

(2) $$(1+\cos\theta )\gamma_{i}^{TOT}=2\left(\sqrt{\gamma_{i}^{LW}\gamma_{I}^{LW}}+\sqrt{\gamma_{i}^{+}\gamma_{I}^{-}}+\sqrt{\gamma_{i}^{-}\gamma_{I}^{+}}\right),$$

where *θ* is the contact angle and *γ ^TOT^* = *γ ^LW^* + *γ ^AB^*.

#### 2.2.3. GNP/PMDS and PDMS Morphology

The surface morphology and the distribution of GNPs into the PDMS matrix were evaluated by scanning electron microscopy (FEI Quanta 400 FEG ESEM/EDAX Genesis X4M microscope; FEI Europe, Eindhoven, The Netherlands).

### 2.3. Antibiofilm Studies

#### 2.3.1. Bacterial Strain and Culture Conditions

The antimicrobial/anti-adhesive activity of GNP/PDMS coatings was assessed using a *Staphylococcus aureus* reference strain (ATCC 25923) and a mCherry-*Pseudomonas aeruginosa* PAO1 strain because these two bacteria are frequently found in implantable medical devices [51]. The mCherry-expressing strain enabled *P. aeruginosa* detection in dual-species biofilms. Bacteria were stored at −80 °C in Luria-Bertani Broth (LB) medium (Thermo Fisher Scientific, Waltham, MA, USA). Before each experiment, both bacteria were spread on Plate Count Agar (PCA; Merck KGaA, Darmstadt, Germany) petri dishes and incubated overnight at 37 °C.

LB broth was then inoculated with individual colonies harvested from PCA plates and incubated overnight at 37 °C, 160 rpm. In the specific case of *P. aeruginosa* cultures, tetracycline antibiotic (1.25 mg L^−1^) was applied to select the transformed bacteria [52]. After centrifugation at 3772× *g*, 18 °C for 10 min (Eppendorf Centrifuge 5810R, Eppendorf, Hamburg, Germany), the pellet was resuspended in fresh LB medium, and a final cell suspension with an optical density at 610 nm of 0.1 (corresponding to 1 × 10^8^ colony-forming units per mL, CFU mL^−1^) was prepared. To form dual-species biofilms, the bacterial suspensions of *S. aureus* and *P. aeruginosa* were mixed in a ratio of 1:1, maintaining the final cell concentration of 1 × 10^8^ CFU mL^−1^.

#### 2.3.2. Antibiofilm Assays

To assess biofilm formation on GNP/PDMS composites, the composites were first sterilized through UV radiation for 1 h. Sterilized surfaces of PDMS, and 1, 2, 3, 4, and 5 wt% GNP/PDMS were placed on the microplate wells (12-well plate, VWR International, Carnaxide, Portugal) and inoculated with 3 mL of the bacterial suspension. Plates were incubated at 37 °C for 24 h under static conditions.

##### Biofilm Quantification

After 24 h of biofilm formation, GNP/PDMS surfaces were detached from the microplate wells, immersed in 2 mL of saline solution, and vigorously agitated for 3 min to obtain biofilm cell suspensions. The number of culturable cells was evaluated by spreading the biofilm suspensions on PCA (after proper dilution in saline solution) followed by CFU counts. The biofilm total and viable cells were assessed by staining biofilm suspensions with the Live/Dead kit (Invitrogen Life Technologies, Alfagene, Portugal) [53] and subsequent analysis in an epifluorescence microscope (Leica DM LB2, Wetzlar, Germany). The number of viable but nonculturable (VBNC) cells was also calculated as the difference between the number of viable and culturable cells [54].

##### Visualization of the Biofilms Using Confocal Laser Scanning Microscopy (CLSM)

The spatial structure of single- and dual-species biofilms of *P. aeruginosa* and *S. aureus* on PDMS and GNP/PDMS surfaces was assessed by CSLM as described by Lima et al. [7]. *S. aureus* and *P. aeruginosa* + *S. aureus* biofilms were first counterstained with 6 µM SYTO^®^9 (Invitrogen Life Technologies, Alfagene, Portugal). All biofilm samples were then observed using a 40× water immersion objective lens (Leica Microsystems, Wetzlar, Germany) in an inverted microscope Leica DMI6000-CS with 488 nm argon and 633 nm helium-neon lasers. Image stacking was acquired with a 1 μm thickness for each sample at a minimum of five random fields. Image processing was performed using the IMARIS 9.1 software package (Bitplane, Zurich, Switzerland) for modelling in two and three dimensions [55]. The CLSM acquisitions were analyzed by the plug-in COMSTAT2 associated with the ImageJ software to determine biofilm structural parameters such as biovolume (µm^3^ µm^−2^) and average thickness (µm) [56].

### 2.4. Statistical Analysis

Statistical analysis was conducted in GraphPad Prism 8 version. After assessing data normality with the Shapiro–Wilk test, the most appropriate tests for mean comparison were applied. The Kruskal–Wallis test was used to evaluate the differences between the contact angles of PDMS, and 1, 2, 3, 4, and 5 wt% GNP/PDMS surfaces, as this variable was not normally distributed. Differences in the number of culturable cells obtained for PDMS, and 1, 2, 3, 4, and 5 wt% GNP/PDMS surfaces were evaluated using one-way analysis of variance (ANOVA). The Mann–Whitney test was applied to evaluate the differences in the number of total, viable, culturable, and VBNC cells between PDMS and 5 wt% GNP/PDMS surfaces, as the variables were normally distributed. Quantitative parameters obtained from confocal microscopy, namely biovolume and thickness, were compared using ANOVA. Statistical significance was indicated as * for *p* < 0.05, ** for *p* < 0.01, *** for *p* < 0.001, and **** for *p* < 0.0001. All experiments were performed in triplicate, and the results presented as mean ± standard deviation (SD) or error (SE).

## 3. Results and Discussion

### 3.1. Graphene Characterization

As particle morphology affects the microstructure and porosity of nanocomposites, the characterization of the graphene sample is of extreme importance [57]. Therefore, the textural properties of GNP powders were first assessed by the N_2_ adsorption-desorption isotherms.

The isotherms of the GNP sample fit the Type-II isotherm profile, characteristic of carbon materials with slit-shaped mesoporosity, according to IUPAC classification [58]. The physisorption isotherms obtained are in accordance with those described in previous reports on pristine GNPs [59,60]. The presence of an H3 type hysteresis loop indicates capillary condensation phenomena and mesoporous materials that comprise aggregates of plate-like particles [61]. The sharp rise of N_2_ uptake at low relative pressure is quite characteristic of microporous materials [62].

The results of N_2_ adsorption-desorption isotherms are reflected in the specific surface area (S_BET_), external surface area (S_meso_), micropore volume (V_micro_), and total pore volume (V_p_) of GNPs, textural parameters that are summarized in Table 1.

As one might expect, the results of N_2_ adsorption-desorption isotherms proved that GNPs present, along with the microporous character (*V*_micro_ of about 0.05 cm^3^ g^−1^), an extended mesoporosity, and a high specific surface area, which is in good agreement with that provided by the manufacturer. The obtained surface area is still significantly lower than the theoretical surface area of individual graphene sheets (2630 m^2^ g^−1^) [63]. However, these results are consistent with the GNP structure, which is characterized by overlapped graphene layers that limit nitrogen adsorption, as previously noted by Srinivas et al. [64]. As it is well known that high surface area potentiates the interaction with bacterial cells and, consequently, cell death [65], the use of these GNPs for antimicrobial applications is a promising approach. Furthermore, several studies reported the potential of microporous materials in the biomedical field for antibacterial applications [66].

### 3.2. Physicochemical Characterization of GNP/PDMS Surfaces

Because the surface properties, such as hydrophobicity and charge, are known to affect the extent of cell adhesion and biofilm formation [67], the GNP/PDMS surfaces were first characterized by contact angle measurements and the calculation of the respective free energy of interaction (ΔG*_iwi_*) among the two entities of a specific surface (*i*) when immersed in water (*w*) (Table 2).

The results showed that tested surfaces presented a hydrophobic behaviour. This evidence is in accordance with previously published studies using graphene-based materials [68,69,70], including pristine GNPs embedded in a PDMS matrix [38]. Although it is not possible to observe a linear trend in the evolution of hydrophobicity with the incorporation of increasing GNP loadings, the enhancement of surface hydrophobicity with the incorporation of 5 wt% is quite remarkable. Indeed, it was possible to verify that 5 wt% GNP/PDMS surfaces showed a higher value for the water contact angle (*p* < 0.0001) compared to PDMS.

**Figure 2:**
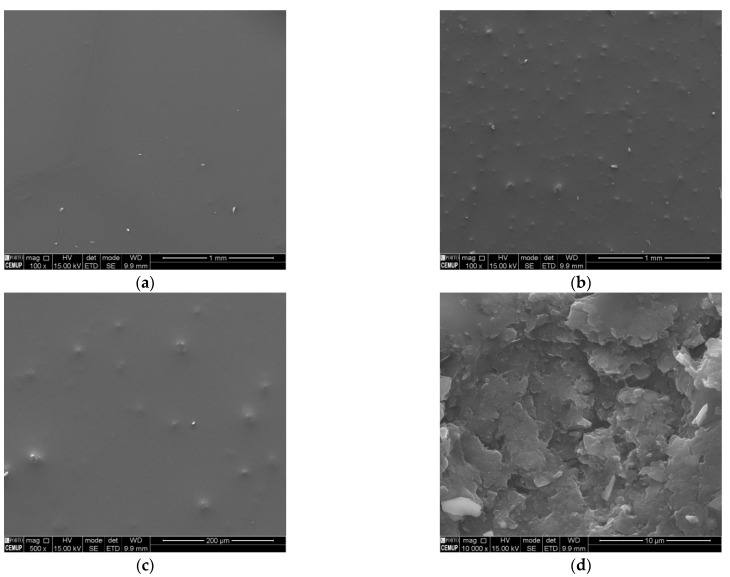
Scanning electron microscopy (SEM) images of (**a**) PDMS (magnification of 100×) and 5 wt% GNP/PDMS surfaces with a magnification of (**b**) 100×, (**c**) 500×, and (**d**) 10,000×.

Considering that the topography of nanocomposite films may significantly influence the cell adhesion behaviour [71], the characterization of 5 wt% GNP/PDMS surfaces was supplemented by SEM analysis (Figure 2).

From the SEM images, it was possible to draw some conclusions regarding the overall incorporation and distribution of GNPs into the PDMS matrix. Although some bigger conglomerates still exist, reflecting the layer-stacked compact structure of GNPs (which results from the strong π-π interactions between graphene nanoplatelets) [72], in general, GNPs were uniformly dispersed into the PDMS network (Figure 2b,c). This dispersion uniformity guarantees a larger exposure of the surface of GNPs to bacterial cells and, potentially, higher antimicrobial activity [28,73]. The typical flake-like structure of GNPs is perfectly evidenced at higher magnifications (Figure 2d).

### 3.3. Biological Characterization

**Figure 3:**
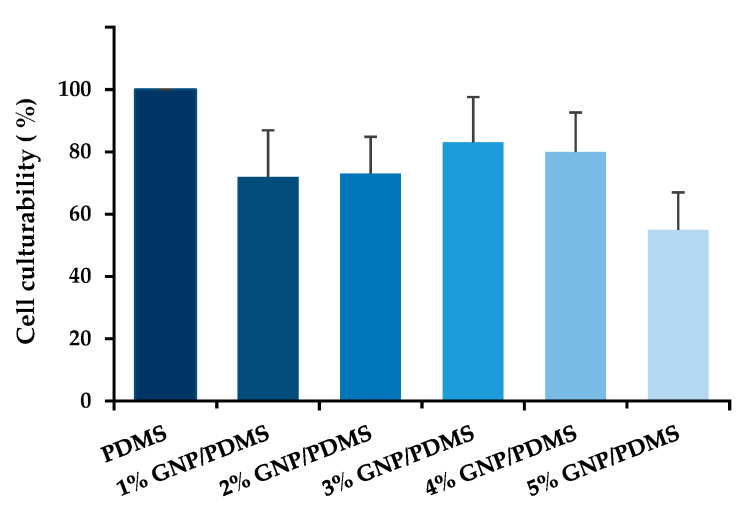
Percentage of *S. aureus* culturable cells after 24 h of biofilm formation on PDMS (control), and 1, 2, 3, 4, and 5 wt% GNP/PDMS composites. The means ± standard error (SE) are presented.

The antibiofilm activity of PDMS surfaces containing different GNP loadings was first assessed against *S. aureus* ATCC 25923. After 24 h of incubation with 1, 2, 3, 4, and 5 wt% GNP/PDMS composites, the culturable cells were quantified (Figure 3). The addition of GNPs to the PDMS matrix resulted in a decrease in cell culturability. A total reduction of up to 55% was achieved for the 5 wt% GNP/PDMS surface, which was chosen for further assays, although there were no statistically significant differences between GNP loadings. These results are in line with those found in the literature, where higher concentrations of graphene derivatives have demonstrated a higher bactericidal or bacteriostatic activity [74,75,76].

The results of *S. aureus* culturability were supported by the contact angle measurements, which showed higher hydrophobicity levels for 5 wt% GNP/PDMS composite materials compared to the other tested surfaces. Some studies reported the efficient use of hydrophobic and superhydrophobic surfaces to reduce bacterial adhesion (including of *S. aureus* and *P. aeruginosa*) and, consequently, biofilm formation [77,78]. The limited antibacterial activity shown by pristine GNP/PDMS composites (reductions of on average 26%; Figure 3), especially those of lower loads, may be explained by the existence of vigorous inter-plane interactions that, by inducing the formation of stronger agglomerates, can reduce the available surface area and, therefore, hinder the GNP mode of action [29].

The 5 wt% GNP/PDMS surface was evaluated in subsequent assays with single- and dual-species biofilms of *S. aureus* and *P. aeruginosa,* where its antibiofilm activity was assessed by means of total, viable, culturable, and VBNC cells quantification, and confocal microscopy. By choosing these specific microorganisms, a better understanding of the GNP antibiofilm performance against Gram-positive (*S. aureus*) and Gram-negative (*P. aeruginosa*) bacteria can be achieved.

**Figure 4:**
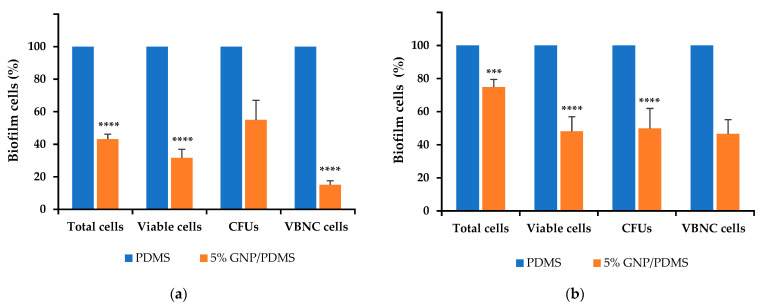
Percentage of *S. aureus* (**a**) and *P. aeruginosa* (**b**) single-species biofilm cells on PDMS (control) and 5 wt% GNP/PDMS composites. The means ± SE are presented. Significant differences compared with PDMS are represented for *p* < 0.001 by *** and *p* < 0.0001 by ****.

The antibiofilm activity of 5 wt% GNP/PDMS surfaces against single-species biofilms of *S. aureus* and *P. aeruginosa* is shown in Figure 4. The analysis of biofilm cells indicated that *S. aureus* biofilm (Figure 4a) grown on 5 wt% GNP/PDMS surfaces presented significant reductions in the number of total (51%), viable (69%), and VBNC cells (85%) compared to PDMS (*p* < 0.0001). However, the reduction achieved for the culturable cells (55% compared to PDMS) was not statistically significant (*p* > 0.05, Figure 3 and Figure 4a).

Regarding *P. aeruginosa* (Figure 4b), biofilms formed on 5 wt% GNP/PDMS surfaces presented lower reduction percentages of total (25%, *p* < 0.001), viable (52%, *p* < 0.0001), and VBNC cells (53%, *p* > 0.05) compared to PDMS. When compared with *S. aureus* biofilms, the same extent of reduction of cell culturability was observed (51% compared to PDMS, *p* < 0.0001).

The antimicrobial and antibiofilm activity of graphene-based nanocomposites against *S. aureus* and *P. aeruginosa* was previously reported in other studies. However, those were studies performed with functionalized graphene-based materials, including graphene oxide [79,80,81,82] or reduced graphene oxide [83,84]. Additionally, although some studies report the antimicrobial use of GNP against common causative microorganisms of IAIs [38,73,85,86,87], to the best of our knowledge, only two publications refer to the antimicrobial activity of GNPs against *S. aureus* and *P. aeruginosa*, and none of these makes use of pristine GNPs incorporated into a surface [88,89]. Concerning the greater efficacy of GNPs against *S. aureus* in comparison to *P. aeruginosa* [81], earlier publications also reported that Gram-positive bacteria are more susceptible to graphene-based materials than Gram-negative bacteria [90,91]. This is mainly due to differences in the cell wall structure [91]. The outer membrane layer in Gram-negative bacteria can act as a protective barrier against graphene exposure compared to Gram-positive bacteria [90].

Overall, these results suggest that loading PDMS with 5 wt% GNPs presents anti-adhesive and antimicrobial properties against both strains, also exerting an effective role in reducing the percentage of VBNC cells, particularly for *S. aureus* (85%). Given the involvement of these cells in the recurrence of infections [54], our results indicate that 5 wt% GNP/PDMS surfaces may be advantageous for application in IMDs. At the same time, as non-oxidized nanoplatelets induce lower ROS production, they can be considered safer materials for use as antimicrobial agents compared with their functionalized derivatives [92].

**Figure 5:**
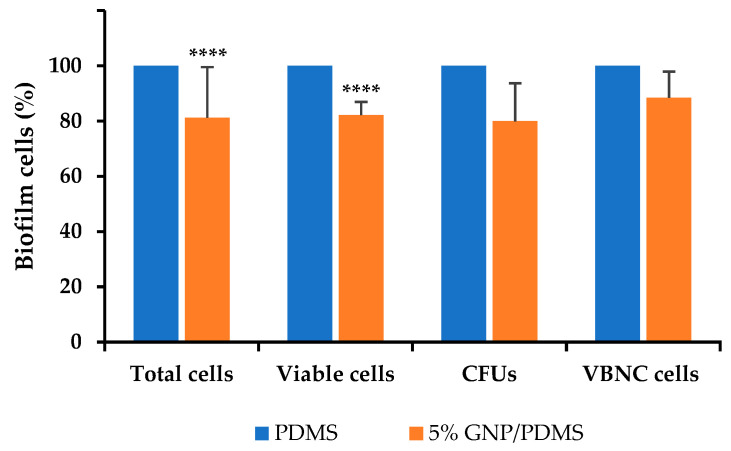
Percentage of *S. aureus* + *P. aeruginosa* biofilm cells on PDMS (control) and 5 wt% GNP/PDMS composites. The means ± SE are presented. Significant differences compared with PDMS are represented for *p* < 0.0001 by ****.

Considering that in the natural environment biofilms are characterized by multi-species communities, and that the investigations carried out so far failed to predict the antibiofilm performance of GNP composites on this scenario, 5 wt% GNP/PDMS surfaces were tested against dual-species biofilms of *S. aureus* and *P. aeruginosa* (Figure 5). It was possible to observe lower reduction percentages of total (24%), viable (23%), culturable (20%), and VBNC cells (29%) compared to PDMS. Therefore, the antibiofilm activity of 5 wt% GNP/PDMS surfaces was higher in single- than in dual-species biofilms. However, there was still a significant reduction in the number of total and viable cells (*p* < 0.0001), hence these GNPs constitute promising materials in this field.

The cell culturability of dual-species biofilms also showed that the dominant strain in co-culture biofilms was undoubtedly the Gram-negative *P. aeruginosa*, with a percentage of 93% and 88% on PDMS and 5 wt% GNP/PDMS composites, respectively (Figure S2a in Supplementary Material). These outcomes are in agreement with previously reported studies, which demonstrated that *P. aeruginosa* inhibits the growth of *S. aureus* in dual-species biofilms [7,93,94]. The higher percentage of *P. aeruginosa* in the co-culture may justify the similarity of behaviours between the single-species biofilms of this Gram-negative bacteria and the mixed ones, which showed higher overall resistance to GNP/PDMS composites.

**Figure 6:**
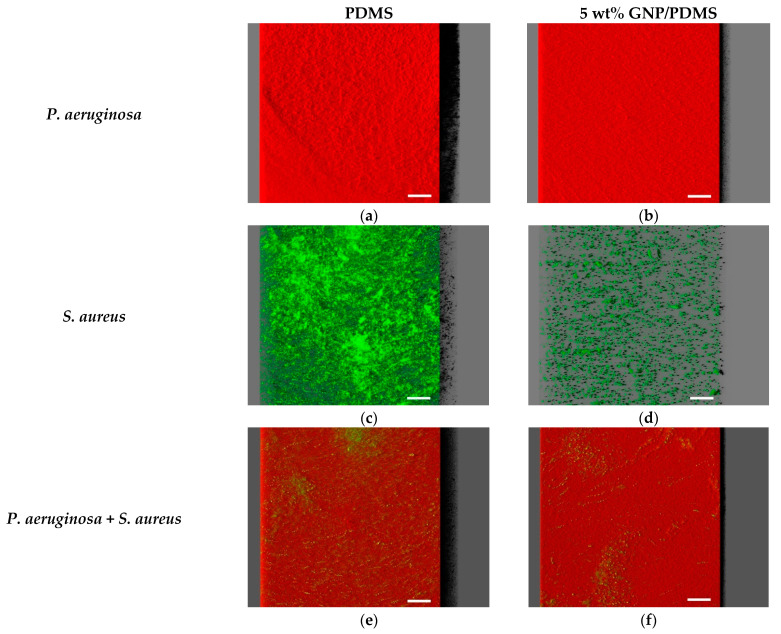
Single-species biofilms of (**a**,**b**) *P. aeruginosa* and (**c**,**d**) *S. aureus*, and (**e**,**f**) mixed biofilms of *P. aeruginosa* (in red) and *S. aureus* (in green) formed on PDMS (left images) and 5 wt% GNP/PDMS (right images). These pictures were extracted from confocal files by IMARIS software and present an aerial view of biofilm structures. The shadow projection on the right represents the biofilm thickness. Scale bars are 50 μm.

Representative CLSM images of single-species biofilms of *P. aeruginosa* and *S. aureus* and dual-species biofilms on PDMS (control surface) and GNP/PDMS composites are shown in Figure 6.

**Figure 7:**
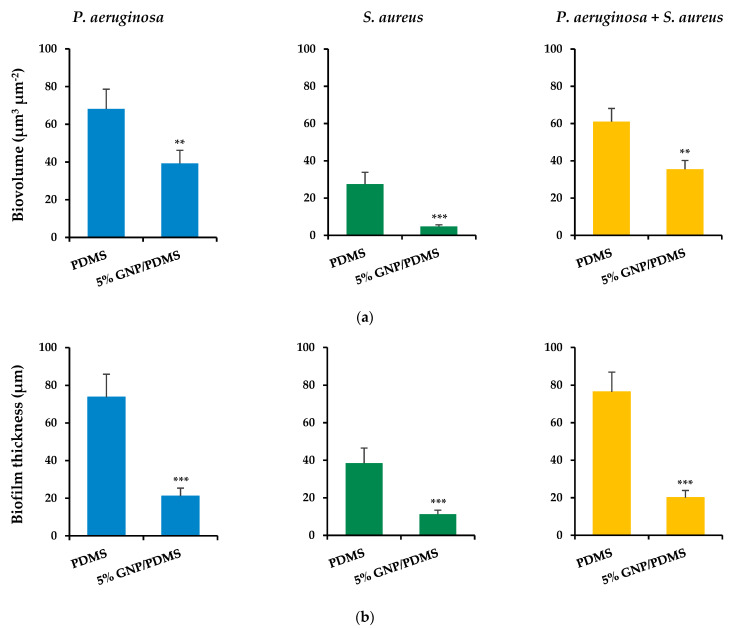
(**a**) Biovolumes and (**b**) thickness of *P. aeruginosa* and *S. aureus* single-species biofilms, and mixed biofilms (*P. aeruginosa + S. aureus*) on PDMS and 5 wt% GNP/PDMS surfaces. Both parameters were extracted from confocal files with the ImageJ software. The means ± standard deviation are presented. Significant differences are represented for *p* < 0.01 by ** and <0.001 by *** when compared with PDMS.

Concerning single-species biofilms, marked variability in the three-dimensional (3D) structure of biofilms could be observed between the bacterial strains after 24 h of biofilm growth, regardless of the surface material tested (Figure 6a–d). *P. aeruginosa* completely covered the surface, developing dense and thick biofilms (Figure 6a,b), whereas the *S. aureus* strain formed highly heterogeneous biofilms consisting of cell aggregates dispersed on the surface (Figure 6c,d). This visual information was supported through the calculated structural parameters of biofilm biovolume and thickness (Figure 7a,b).

Indeed, *P. aeruginosa* formed biofilms which had on average 74% more biovolume and were 47% thicker than staphylococcal biofilms. Looking at the surface effect, the PDMS surfaces showed the highest biofilm amount and thickness (shadow projection on the right of Figure 6a,c) when compared to the graphene-based surface (Figure 6b,d). In fact, the GNP/PDMS composite was able to reduce the *P. aeruginosa* and *S. aureus* biovolume and biofilm thickness compared to the PDMS surface (*p* < 0.01, Figure 7a,b). However, whereas the decrease in biofilm thickness was similar for both strains (approximately 71%), the biovolume reduction on the surface containing graphene was higher for *S. aureus* than for *P. aeruginosa* (83% versus 42% reduction). This result is in agreement with that obtained by epifluorescence microscopy (Figure 4), in which the reduction in the total cell number between the PDMS and 5 wt% GNP/PDMS surface was 57% for *S. aureus* biofilms and only 25% for *Pseudomonas* biofilm.

Figure 6e,f shows the architecture of dual-species biofilms of *P. aeruginosa* and *S. aureus* grown on PDMS and 5 wt% GNP/PDMS surfaces, respectively. Dual-species biofilms were quite dense and thick as those formed by *P. aeruginosa* alone (Figure 6a,b). Nevertheless, as for the single-species biofilms (Figure 6a–d), less biofilm amount was observed on the graphene composite compared to PDMS. The quantitative results confirmed the similarity of biovolume and thickness values between dual-species biofilms and *P. aeruginosa* biofilms (Figure 7), regardless of the tested surface. Additionally, biofilms developed on GNP/PDMS had 42% less biovolume and 74% less thickness than on PDMS coating, reinforcing the anti-adhesive and antimicrobial activities of this surface in typically more adverse conditions such as the presence of different microbial species.

The CLSM study also revealed that the dominant strain in co-culture biofilms was clearly the Gram-negative bacterium *P. aeruginosa* (Figure 6e,f, and Figures S1 and S2b in Supplementary Material). A small number of *S. aureus* cells were heterogeneously distributed across surfaces (Figure 6e,f and Figure S1 in Supplementary Material), and the percentage of *S. aureus* population in biofilms was approximately 11% (Figure S2 in Supplementary Material). This suggests a strong antagonistic behavior by *P. aeruginosa* towards the Gram-positive bacterium *S. aureus*. In terms of vertical distribution within biofilms, *S. aureus* was relatively more abundant at the top, whereas the bottom layers of the biofilm consisted predominantly of *Pseudomonas* cells (Figure S2 in Supplementary Material).

This work demonstrates the antimicrobial activity of GNP/PDMS surfaces against single- and dual-species biofilms of *S. aureus* and *P. aeruginosa*. Although the studied pristine GNP-based materials combined good performance with the simplicity of preparation compared to functionalized composites, graphene functionalization with bioactive molecules and/or polymers may improve its antimicrobial performance. This will be explored in the future, as the percentages of biofilm reduction achieved here were lower than those reported in the literature for these particular bacteria in other coating systems for implantable medical devices [95]. For example, using nanocoatings based on magnetite, polyethyleneglycol, and a biologically active molecule (polymyxin B-PM), Caciandone and colleagues [95] were able to reduce *S. aureus* and *P. aeruginosa* culturable cells of up to four and five orders of magnitude, respectively.

Considering the ultimate goal of the produced GNP/PMDS coatings, cytotoxicity assays will be performed in the near future. However, it is known that, although graphene-based materials are not devoid of risks for the human body, they possess lower toxicity and higher biocompatibility than other carbon allotropes, including carbon nanotubes [96]. Furthermore, the vast majority of the studies in this field have shown that conjugating graphene with other biocompatible materials can help to create graphene materials with reduced bioaccumulation impact [97].

## 4. Conclusions

In this work, GNP/PDMS surfaces were produced and tested for the prevention of single- and dual-species biofilms of *S. aureus* and *P. aeruginosa*. It was shown that GNP/PDMS surfaces present high hydrophobicity and a good dispersion level, which are promising characteristics for the application of this carbon compound as an antibacterial agent.

Furthermore, biological assays showed that 5 wt% GNP loading was able to reduce the total and viable cell numbers of *S. aureus* and *P. aeruginosa* biofilms compared to PDMS. Although dual-species biofilms demonstrated higher resistance to the antimicrobial activity of 5 wt% GNP/PDMS surfaces, the performance of these coatings in inhibiting single- and dual-species biofilm cells was supported by confocal microscopy analysis, with a significant decrease in the two measured quantitative parameters—biofilm biovolume and thickness. Overall, the results obtained present enough potential to envisage a possible application of 5 wt% graphene/PDMS as an antimicrobial coating in biomedical devices. Future investigations are needed to evaluate the biocompatibility of these specific graphene derivatives, due to the lack of studies in the field.

## Figures and Tables

**Table 1:** Textural properties of graphene nanoplatelets (GNPs).

Sample	*S*_BET_ (m^2^ g^−1^)	*S*_meso_ (m^2^ g^−1^)	*V*_micro_ (cm^3^ g^−1^)	*V*p *p*/*p*_0_ = 0.99 (cm^3^ g^−1^)
GNPs	464	363	0.045	0.535

**Table 2:** Contact angles with water (*θ_w_*), formamide (*θ_F_*), and α-bromonaphthalene (*θ_B_*) for bare polydimethylsiloxane (PDMS) and GNP/PDMS surfaces. The respective free energy of interaction (Δ*G_iwi_*) is also included.

Surface	Contact Angle (°)			Hydrophobicity (mJ m^−2^)
	*θ_w_*	*θ_F_*	*θ_B_*	Δ*G_iwi_*
PDMS	110.2 ± 3.6	112.4 ± 3.1	90.5 ± 4.9	−50.2
1 wt% GNP/PDMS	108.6 ± 2.2	104.1 ± 3.3	82.5 ± 3.5	−58.9
2 wt% GNP/PDMS	110.4 ± 1.9	105.7 ± 4.5	88.2 ± 3.6	−63.1
3 wt% GNP/PDMS	110.3 ± 1.6	105.5 ± 2.7	88.6 ± 3.1	−57.2
4 wt% GNP/PDMS	111.9 ± 2.3	107.9 ± 5.7	92.8 ± 2.8	−65.5
5 wt% GNP/PDMS	121.8 ± 3.3	113.9 ± 3.6	102.4 ± 4.1	−87.7

## Data Availability

The data presented in this study are available on request from the corresponding authors. The data are not publicly available yet as some datasets are being used for additional publications.

## Supplementary Materials

The following supporting information can be downloaded at: https://www.mdpi.com/article/10.3390/nano12030355/s1, Figure S1: Spatial heterogeneity of dual-species biofilms of *P. aeruginosa* (labeled by the red fluorescent protein mCherry) and *S. aureus* (countermarked in green with Syto9) formed on 5 wt% GNP/PDMS: section views of the CLSM image presented in Figure 6f. Dotted white lines indicate vertical sections. Scale bar is 100 μm; Figure S2: Proportion of *S. aureus* (in blue) and *P. aeruginosa* (in orange) (a) culturable cells and (b) biovolume in dual-species biofilms formed on GNP/PDMS surfaces.
