# Supplementary material for: Performance of Graphene/Polydimethylsiloxane Surfaces against S. aureus and P. aeruginosa Single- and Dual-Species Biofilms
Source: Nanomaterials (Basel). 2022 Jan 22;12(3):355. doi: 10.3390/nano12030355 (PMC8839372; doi:10.3390/nano12030355)
Supplement: Supplementary file 1 [file nanomaterials-12-00355-s001.zip › nanomaterials-1556672-supplementary.pdf]

Supplementary material

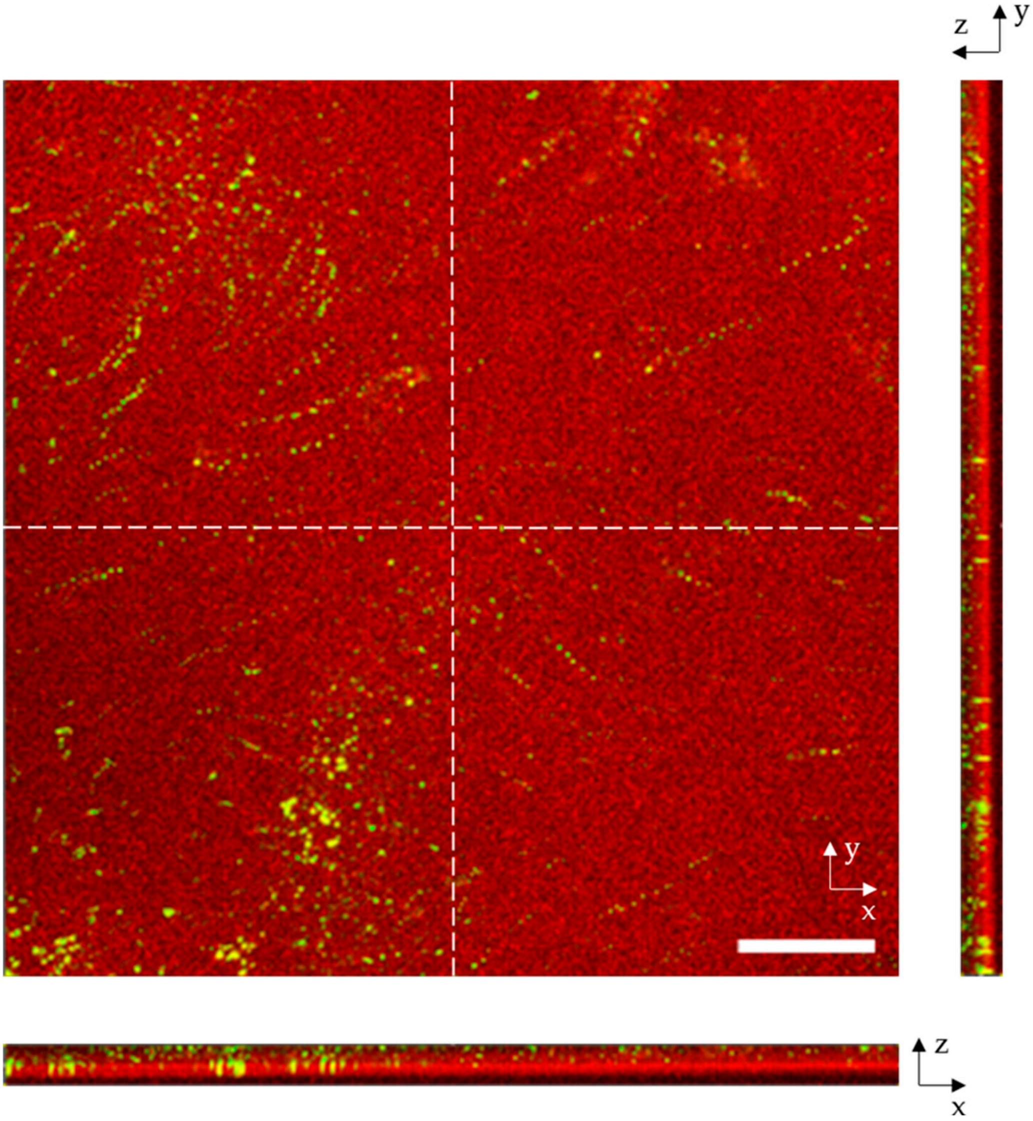

Figure S1: Spatial heterogeneity of dual-species biofilms of *P. aeruginosa* (labeled by the red fluorescent protein mCherry) and *S. aureus* (countermarked in green with Syto9) formed on 5 wt% GNP/PDMS: sections views of the CLSM image presented in Figure 6f. Dotted white lines indicate vertical sections. The scale bar is 100 µm.

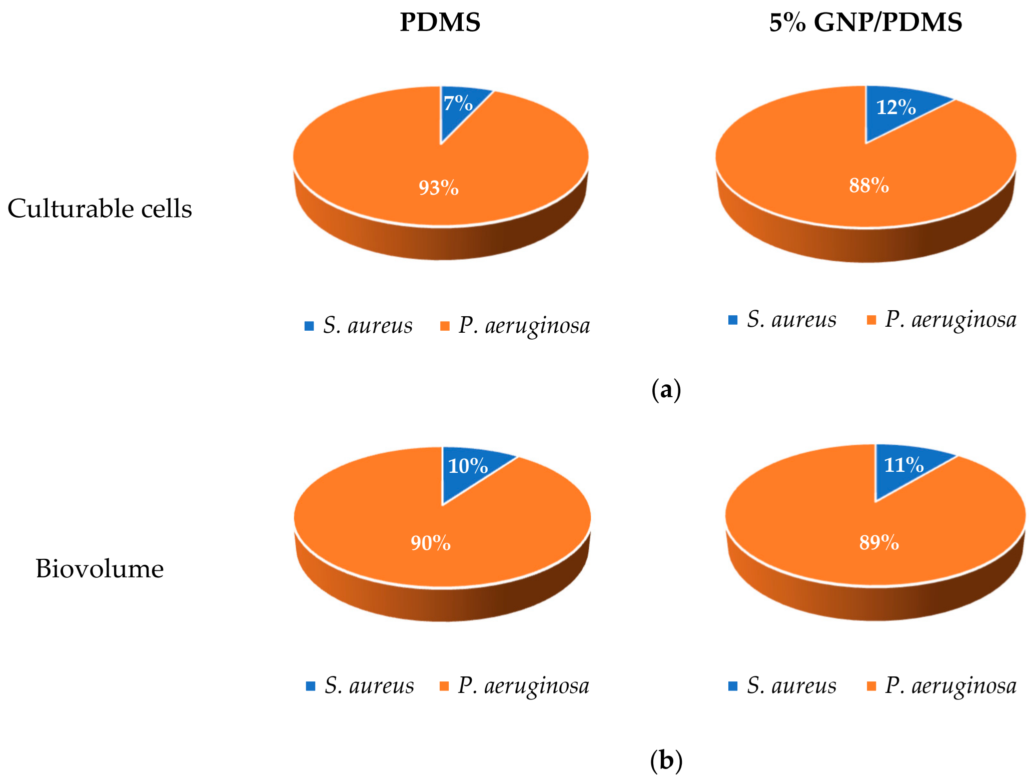

Figure S2: Proportion of *S. aureus* (in blue) and *P. aeruginosa* (in orange) (**a**) culturable cells and (**b**) biovolume in dual-species biofilms formed on 5 wt% GNP/PDMS surfaces.
